# The role of diagnostic ureteroscopy in the era of computed tomography urography

**DOI:** 10.1186/s12894-015-0068-8

**Published:** 2015-07-25

**Authors:** Shay Golan, Andrei Nadu, David Lifshitz

**Affiliations:** Institute of Urology, Rabin Medical Center, Petah Tikva, and Sackler Faculty of Medicine, Tel Aviv University, Tel Aviv, Israel

**Keywords:** Upper urinary tract urothelial carcinoma, Ureteroscopy, Computed tomography urography

## Abstract

**Background:**

To examine the contemporary role of ureteroscopy in the diagnosis of upper urinary tract urothelial carcinoma.

**Methods:**

We retrospectively evaluated 116 diagnostic ureteroscopies, performed in our institution to rule out primary UTUC. Demographics, cytological findings and interpretation of preoperative imaging were obtained. Ureteroscopic diagnosis and histological results were recorded and the predictive values of diagnostic studies were determined. Follow-up data was reviewed to evaluate the oncological outcomes in patients treated endoscopically.

**Results:**

The pre-ureteroscopic evaluation included CTU in 91 (78 %) patients. Positive and Negative predictive values of CTU were 76 and 80 %, respectively. Typical filling defect on CTU was demonstrated in 38 of 89 patients. UTUC has been ruled out in 9 patients (24 %) with suspicious filling defect on CTU. Endoscopic approach was implemented in 7 patients (18 %). During a median follow up period of 17 months (IQR, 9–25) none of the followed patients experienced disease progression.

**Conclusions:**

Nephroureterectomy was spared from 42 % of patients who underwent diagnostic ureteroscopy for suspected UTUC, demonstrated on CTU. In about half of those patients tumor has been ruled out and the others were managed endoscopically. Therefore, diagnostic ureteroscopy is advised as a crucial step in confirming UTUC and treatment planning.

## Background

Upper urinary tract urothelial carcinoma (UTUC) is an uncommon malignancy, accounting for ~5 % of urothelial tumors [1]. The diagnosis of UTUC can be challenging, requiring a combination of radiographic, cytologic and endoscopic means. Time honored radiological tools such as intravenous urography and retrograde uretropyelograpy are currently replaced by modern computerized tomography urography (CTU) [2]. Diagnostic ureteroscopy is often performed following CTU. Flexible ureteroscopy allows exploration of the upper urinary tract and is beneficial when diagnostic uncertainty exists. It has the advantages of offering direct view of the tumor, ruling out other pathologies and achieving tissue diagnosis. Although nephrouereterectomy is considered the gold standard treatment of UTUC, endoscopic ablation and resection of the tumor can be successfully utilized in selected cases based on tumor size and histology, as determined during ureteroscopoy. In low grade low volume tumors endoscopic management provides cancer related and overall survival equivalent to that of nephroureterectomy [3]. Despite the above, the routine use of ureterosocpy following CTU is controversial. Ureteroscopy is an invasive procedure that may be associated with morbidity as well as a potential risk of tumor seeding [4, 5]. In the last edition of “Campbell’s urology” the authors do not support a routine ureteroscopic confirmation of UTUC [6], while the European guidelines on UTUC (2015 version) advocate the use of diagnostic ureteroscopy with biopsy, especially in cases where additional information will impact treatment decisions. The purpose of this study was to evaluate the diagnostic value of ureteroscopy in patients who underwent workup for suspected UTUC and to assess the impact of ureteroscopy on the management of UTUC.

## Methods

This study was approved by “Rabin Medical Center” ethics committee. Between 2003 and 2010, 1818 ureteroscopies, were performed at our institution, of which 116 (6.3 %) were diagnostic, aimed to rule out primary UTUC. The indications for diagnostic ureteroscopy included painless hematuria with positive urinary cytology and negative cystoscopy or imaging findings that may indicate UTUC. CTU was not performed in patients with chronic renal failure (eGFR < 30 mL/min/1.73 m^2^) or severe contrast allergy. Although ultrasound is not accepted as a standard modality for UTUC investigation, we reported on ultrasound results when it was available (either performed as first imaging modality in the community or in cases of chronic renal failure or severe contrast allergy). The medical chart of each patient was reviewed to obtain demographics, cytological findings and the interpretation of preoperative imaging. Because of the retrospective design of this study our institutional ethics committee waived the need for written informed consent from participants.

Complete endoscopic examination of the ureter, renal pelvis and calyx has been performed in all patients. Ureteroscopy was performed following a retrograde study, using 8FR rigid ureteroscope (Wolf), advancing the instrument as much as possible. Renal inspection was performed using flexible ureteroscope (DUR-8 (ACMI) or in later years the Flex- X™ (Stortz)). Biopsy forceps or stone collection basket were used to obtain tissue from suspected lesion. Tumor grade and stage were assigned using the world health organization classification [7] and the 6th edition of the AJCC/UICC TNM staging system [8] by specialized pathologists at our institution. Patients with histologically confirmed UTUC were referred to nephroureterectomy usually within one month following the diagnostic procedure or, in selected cases, managed conservatively with endoscopic resection. Patients with low grade, <1 cm UTUC were eligible for endoscopic treatment. Holmium laser and a bugbee electrode were utilized for tumor resection and ablation.

Intra- and peri-operative complications were reviewed and staged according to Clavien-Dindo classification [9]. The first follow-up ureteroscopy was preformed 3 month after endoscopic tumor resection. Thereafter, patients were followed with ureteroscopy or CTU, alternately, every 3 months during the first two years. Patients with chronic kidney disease were referred to a nephrologist for consultation. Follow-up data was reviewed to include the oncological outcomes (disease recurrence and progression) of patients managed with nephron sparing endoscopic approach.

The diagnostic value (positive and negative predictive values) of urine cytology, ultrasound and CTU was determined. Positive cytology was defined as malignant cells or atypical cells, highly suggestive of urothelial carcinoma. Intraluminal Soft-tissue mass, demonstrated by ultrasound, or filling defect in contrast opacified collecting system, demonstrated by CTU, were considered positive for UTUC. The endoscopic appearance, supported by histologic examination, when available, served as the standard of reference.

## Results

### Clinical characteristics and diagnostic studies

The study cohort included 116 patients who underwent diagnostic ureteroscopy between November 2003 and December 2010. Demographics and clinical characteristics of the study patients are summarized in Table 1. Half (51 %) of the patients reported at least one episode of gross hematuria and 35 % had a history of lower urinary tract urothelial carcinoma. The preureteroscopic evaluation included urine cytology, ultrasound and CTU in 91 (78 %), 84 (72 %) and 89 (77 %) patients, accordingly. Results of all three studies were available in 47 patients (40.5 %). CTU was not performed in 22 patients with chronic renal failure and 3 patients with severe contrast allergy. Detailed description of the findings, according to the performed study is presented in Table 2. Visual diagnosis of UTUC was made in 47 patients (40 %), supported by histology in 38 of them (80 %). A reliable histological report could not be obtained in 9 patients due to insufficient amount of tissue or technical artifacts.

**Table 1:** Demographic and clinical patient characteristics

Characteristic	*N = 116*
Age, years	
Median (IQR)	70.5
Mean (range)	68 (16–90)
Patient gender *n*, (%)	
Female	38 (33)
Male	78 (67)
Hematuria *n*, (%)	
Macroscopic	59 (51)
Microscopic^a^	88 (76)
History of LUTUC *n*, (%)	
None	75 (65)
Low grade	24 (21)
High grade	17 (14)

*LUTUC* Lower urinary tract urothelial carcinoma

^a^ Defined as three or more red blood cells per high power microscopic filed on urinary sediment

**Table 2:** Results of preoperative evaluation - cytological, sonographic and radiographic findings

Method	
Urine Cytology (*n*, %)	
Atypia	26 (22)
Dysplasia	10 (9)
Normal	55 (48)
N/A	25 (21)
Ultra-Sound (*n*, %)	
Intra-luminal mass	9 (9)
Dilation	43 (37)
No finding	32 (27)
N/A	32 (27)
CTU (*n*, %)	
Filling defect	38 (33)
Wall thickening	14 (12)
Dilation	39 (33)
External mass	5 (4)
No finding	12 (10)
N/A	27 (22)

Of the 38 patients, 27 (71 %) had low grade disease and 11(29 %) had high grade disease on biopsy. In 24 cases tumor stage was determined. Ta, T1 and T2 were assigned in 21 (87 %), 2 (8 %) and 1 (5 %) cases, respectively.

All complications observed in this study were Clavien grade I or II. Intraoperative complications included contrast extravasation, observed during ureteroscopy, in 4 patients (3 %). All patients were managed with ureteral stent for one week with no clinical sequel. Febrile UTI and renal colic were observed after ureteroscopy in 7 (6 %) and 4 (3 %) patients, respectively. Conservative treatment was successfully applied in all cases.

### Correlation with ureteroscopy findings

Positive urinary cytology, reported in 36 of 91patients, included atypia in 26 and dysplasia in 10 patients. The calculated PPV and NPV for UTUC were 47 and 58 %, accordingly. Sonographic appearance of intraluminal mass in 9 patients (9 %), yielded PPV and NPV of 89 and 65 %. Typical filling defect, demonstrated during the excretory phase of CTU, was described in 38 of 89 patients. PPV and NPV of CTU were 76 and 80 %, respectively. Considering “wall thickening” as a positive result, increased the NPV to 90 % but decreased the PPV to 67 %. In 4/39 patients with confirmed UTUC no “filing defect” or “wall thickening” was demonstrated on CTU. Three of these patients had Ta low grade tumors in the ureter and one patient had carcinoma *in situ* of the renal pelvis. Table 3 summarizes the association between the results of preoperative studies and ureteroscopic diagnosis.

**Table 3:** Correlation of preoperative studies with the ureteroscopic results

Preoperative findings	Ureteroscopic results		
	Positive for UTUC	Negative for UTUC	Total
*CTU*			
Positive	29	9	38
Negative	10	41	51
Total	39	50	89
*Ultrasound*			
Positive	8	1	9
Negative	26	49	75
Total	34	50	84
*Urine cytology*			
Positive	17	19	36
Negative	23	32	55
Total	40	51	91

### Filling defect on CTU – ureteroscopic results and therapeutic outcomes

UTUC had been confirmed during URS in 29 of 38 (76 %) patients with characteristic filling defect on CTU. 7 patients had high grade disease on biopsy, including: stage T1 in 4, T2 in 1 and non- determined stage in 2 patients. 20 patients had Ta low grade and in 2 patients neither stage nor grade could be determined. UTUC has been ruled out in 9 patients (24 %). Mucosa fold, small extraluminal mass and parapelvic hyperdense cyst and bullous mucosal edema of the renal pelvis were found in 4 patients and no findings, explaining the filling defects, were reported in the remaining 5 patients. No additional intervention was required in these cases.

Patients with UTUC were subjected to nephroureterectomy or endoscopic resection according to tumor characteristics (see above). Two patients, found to have metastatic disease, were referred to cisplatin based chemotherapy.

**Fig. 1:**
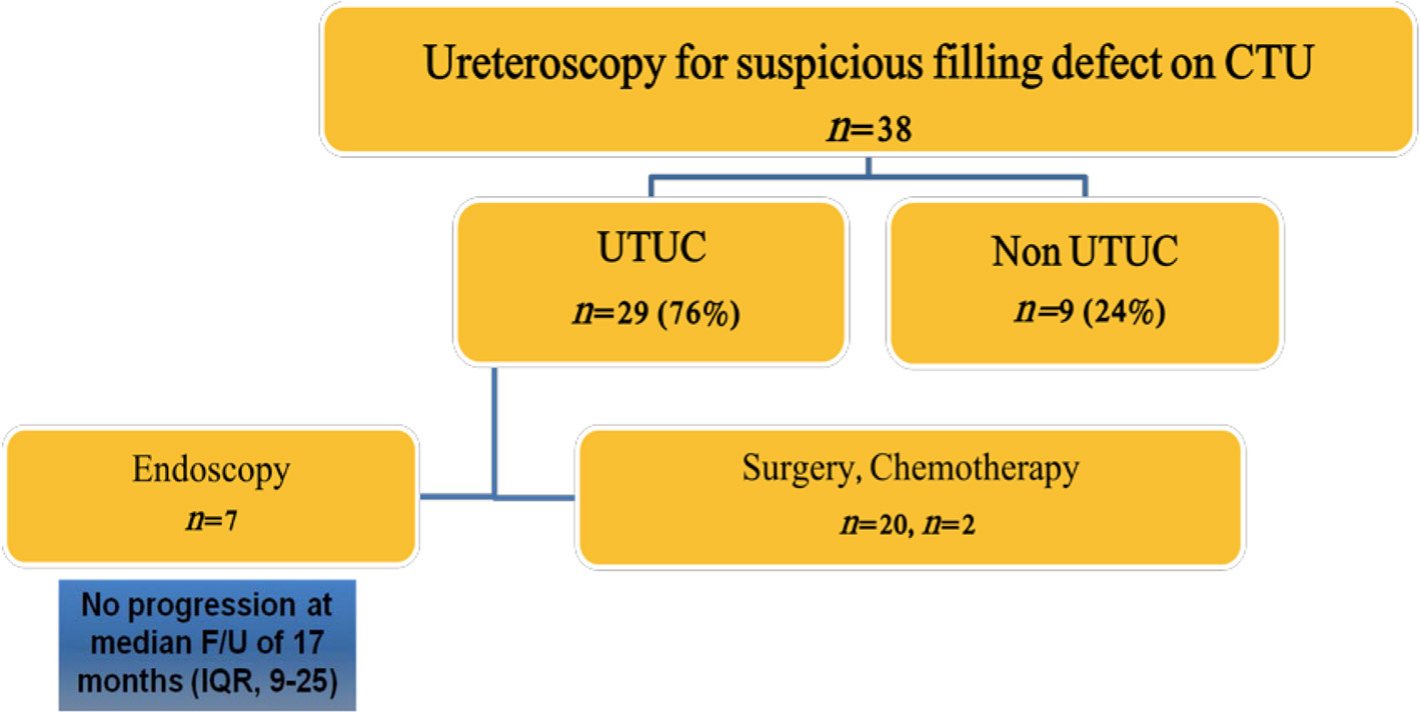
Diagnostic-Therapeutic Flowchart of patients with filling defect on pre-ureteroscopic CTU

Overall, 20 patients underwent nephroureterectomy while nephron sparing endoscopic approach was implemented in 7 patients who underwent tumor resection with continues surveillance. One of these patients was lost to follow-up. During a median follow up period of 17 months (IQR, 9–25) none of the 6 followed patients experienced disease progression. Tumor recurrence was diagnosed and treated successfully in 2 patients, 5 and 14 months after initial resection (Fig. 1).

## Discussion

CTU is currently the recommended imaging modality for evaluation of the upper urinary tract. With ongoing radiological improvements, the added value of diagnostic ureteroscopy is not always clear. We examined the current role of ureteroscopy in the diagnosis of urothelial carcinoma. We found that nephroureterectomy was spared from 42 % (16/38) of patients who underwent ureteroscopy for presumptive UTUC, based on CTU. UTUC was ruled out in 9 patients and treated endoscopically in 7 patients. In the nephron sparing group we observed no disease progression during short term follow-up.

Although UTUC is relatively rare, its incidence has increased in the last decades [10, 11]. One possible explanation for this trend is increased use of CTU, a sensitive tool to detect upper urinary tract abnormalities [2]. The ability of CTU to demonstrate small tumors is well accepted but at the same time, growing use of CTU may increase the detection of benign findings. The positive predictive value (PPV) of CTU for the detection of UTUC is variable, depends on CTU technique and has been reported to be moderate (50-80 %) [12–15]. Intra- and extra-luminal conditions may simulate UTUC on CTU. Inflammatory changes, debris or blood clots may mimic intraluminal tumor. Indentation by extraluminal mass or vascular malformation may also cause false positive results [12]. In our study, the PPV of CTU was 80 %. Non-UTUC findings (mucosa fold, small extraluminal mass and parapelvic hyperdense cyst and bullous mucosal edema) were found during ureteroscopy in 4/9 cases and normal upper urinary tract was described in 5/9 cases. None of these patients had positive urinary cytology. Sadow *et al.* reported on extremely low PPV in small (≤5 mm) upper urinary tract filling defects on CTU [14]. All 17 cases of small filling defects in their study were found to be false positive. In accordance, the five completely normal ureteroscopies in our study were performed in patients with small filling defects on CTU.

The treatment options for confirmed UTUC include nephroureterectomy or, in selected patients, nephron sparing procedure. In the last two decades endoscopic management of UTUC has been extended from patients with imperative indications (chronic kidney disease, bilateral disease or solitary kidney) to selected patients with elective indications. In a review article, Cutress *et al.* reported that the long term renal preservation rate in patients treated endoscopically is 80 % [16]. This information is highly important when considering the potential cardiovascular and overall survival benefits of renal function preservation [17]. Recently published, long term retrospective studies showed that low grade UTUC can be managed endoscopically with cancer related and overall survival equivalent to that of nephroureterectomy [18–20]. Because tumor grade is a significant factor, determining the oncological outcomes of UTUC in patients treated endoscopically, ureteroscopic biopsy is of paramount importance. In our study, 7/38 patients with presumptive UTUC on CTU were treated endoscopically. All had biopsy confirming low grade UTUC less than 1 cm in size. Recurrence occurred in two patients and was amenable for repeat endoscopic resection. No progression was observed during a median follow-up time of 17 months. All complications observed in this study were Clavien grade I or II. Febrile UTI and renal colic were the only post operative complications, reported in 7 (6 %) patients and 4 (3 %) patients, respectively. All patients were successfully treated with antibiotics and analgesics. The potential adverse oncological effect that may be related to delayed nephroureterectomy was beyond the scope of our study, but was addressed in previous publications. No difference in 5 years metastasis-free, cancer specific or overall survival was found in patients who underwent diagnostic ureteroscopy prior to nephroureterectomy compared with patients who did not undergo ureteroscopy [21–23].

Our study is undoubtedly limited by its retrospective design and small size. By the reliance on imaging interpretation, CTU technique was not uniform and the results were subjected to inter observer bias. Despite that, the PPV of CTU in our study is in accordance with results of previous studies [12–15]. Another limitation is the relatively short follow up period of patients treated endoscopically. As mention above, high rate of renal preservation and favorable oncological outcomes were observed in long term studies when patients were carefully selected. This is also reflected by the European guidelines on UTUC, which recognize the option of endoscopic treatment in low grade low stage UTUC [24].

## Conclusions

In this retrospective study, nephroureterectomy was spared from 42 % of patients with presumptive UTUC, demonstrated on CTU. In about half of those patients UTUC has been ruled out and the others were managed endoscopically. Although false positive seems to occur mainly in small filling defects on CTU, there is no room for diagnostic error when nephroureterectomy is being considered. Hence, our study favors ureteroscopy as a crucial step in confirming UTUC and treatment planning.

## Abbreviations

UTUC: Upper tract urothelial carcinoma
CTU: Computerized tomography urography
PPV: Positive predictive value
NPV: Negative predictive value
